# Use of Cerebrospinal Fluid Biomarkers in Diagnosis and Monitoring of Rheumatoid Meningitis

**DOI:** 10.3389/fneur.2019.00666

**Published:** 2019-06-26

**Authors:** Mette Scheller Nissen, Anna Christine Nilsson, Jonatan Forsberg, Jesper Milthers, Martin Wirenfeldt, Christian Bonde, Keld-Erik Byg, Torkell Ellingsen, Morten Blaabjerg

**Affiliations:** ^1^Department of Neurology, Odense University Hospital, Odense, Denmark; ^2^Department of Clinical Research, University of Southern Denmark, Odense, Denmark; ^3^Department of Clinical Immunology, Odense University Hospital, Odense, Denmark; ^4^Department of Pathology, Odense University Hospital, Odense, Denmark; ^5^Department of Neurosurgery, Odense University Hospital, Odense, Denmark; ^6^The Rheumatology Research Unit, Department of Rheumatology, Odense University Hospital, Odense, Denmark

**Keywords:** rheumatoid meningitis, inflammation, anti-CCP, CXCL13, biomarker

## Abstract

Rheumatoid meningitis is a rare extra-articular manifestation of rheumatoid arthritis, often with non-specific symptoms. In most cases brain MRI shows a patchy lepto- and pachymeningeal enhancement, but the diagnosis currently relies on examination of a meningeal biopsy with presence of plasma cells and rheumatoid noduli. Presence of IgM rheumatic factor (RF) has been found in several cases and recently four cases have shown high titer anti-cyclic citrullinated peptide (anti-CCP) in CSF, suggesting this as a potential marker for rheumatoid meningitis. We present a 62 year-old woman with sero-positive (IgM RF and anti-CCP) rheumatoid arthritis, presenting with headache and gait impairment. Brain MRI revealed the classical patchy meningeal enhancement and the diagnosis of rheumatoid meningitis was confirmed by neuropathological examination of a meningeal biopsy. Analysis of the CSF revealed positive IgM RF (92.7 IU/mL) and strongly positive anti-CCP (19,600 IU/mL) and CXCL-13 (>500 ng/L). After treatment with high-dose steroid and Rituximab the clinical symptoms resolved. A 6 month follow-up analysis of CSF showed a dramatic decrease in all these markers with negative IgM RF and a decrease in both anti-CCP (64 IU/mL) and CXCL-13 (<10 ng/L). Our case further underlines the potential use of CSF anti-CCP and IgM RF in the diagnosis of RM and the use of these markers and CXCL-13 in evaluation of treatment response. A case review of 48 cases of rheumatoid meningitis published since 2010, including, symptoms, serum, and CSF findings, treatment, and outcome is provided.

## Background

Rheumatoid meningitis (RM) is a rare but potentially aggressive extra-articular manifestation of rheumatoid arthritis (RA) involving both pachy- and leptomeninges (1, 2). It can occur at all disease stages, and manifestations are often non-specific, mimicking a variety of neurological disorders, malignancies, or infections (1–6). Brain MRI with patchy leptomeningeal contrast enhancement and cerebrospinal fluid (CSF) rheumatoid factor (RF) are useful to guide, but diagnosis still relies on pathological examination of a meningeal biopsy often showing unspecific inflammation, rheumatic noduli, and in some cases vasculitis (2, 7–11). Four recent cases have shown presence of CSF anti-cyclic citrullinated peptide (anti- CCP) in patients with RM (12–15). Here, we describe a patient with RM with strongly positive anti-CCP, IgM RF, and chemokine (C-X-C motif) ligand 13 (CXCL13) levels in CSF that normalized after treatment suggesting a potential use of these markers in both diagnosis and treatment management of RM. Furthermore, we review 48 cases of RM published in the English literature since 2010 focusing on symptoms, serum and CSF findings, treatment, and outcome.

## Case Presentation

A 62 year-old woman was admitted after 4 months history of intermittent frontal headache, nausea, and gait and balance disturbances. She had a 3 year history of IgM-RF and anti-CCP positive RA, with a previously episode of pleuritis. Within the last year, she had been treated with Leflunomide, Infliximab, and was currently treated with Methotrexate and Salazopyrine entabs. Neurological examination was normal, except for a mild gait ataxia and her RA was well-controlled with no symptoms of active synovitis at time of admission.

**Figure 1:**
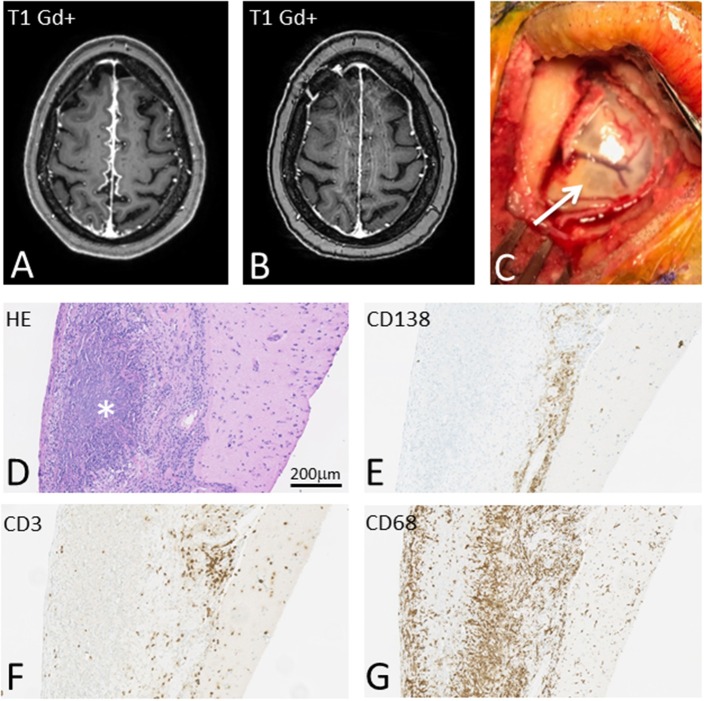
T1-weighted brain MRI showing interhemispheric leptomeningeal Gd+ enhancement before **(A)** and after **(B)** treatment with high dose steroids, Methotrexate and Rituximab. On gross inspection meninges appear severely inflamed **(C)** and pathological examination reveals massive meningeal granulomatous inflammation **(D)** with pre-dominant CD138 positive plasma cells **(E)**, but also CD3 positive T cells **(F)**. Massive infiltration with CD68 positive histiocytes with rheumatic granuloma formation was also seen **(G)**.

Due to chronic headache a brain MRI was performed. This showed patchy interhemispheric pachy- and leptomenigeal enhancement adjacent to the parietal- and occipital lobes (Figure 1A). Blood tests revealed signs of inflammation with high levels of IgM RF (56 IU/mL), anti-CCP (>1,600 U/mL), Interleukin-2 receptor (ILR-2–1,065 kU/L) (Table 1), c-reactive protein (43 mg/L), and erythrocyte sedimentation rate (106 mm). Remaining systemic antibody examinations were negative (anti-DNA antibody, anti-nuclear antibody (ANA) IgG, anti-neutropil cytoplasmatic antibody (ANCA) IgG, Anti-Ro (SSA)/La (SSB), anti-cardiolipin antibody, phospholipid antibody, and lupus anticoagulant). Immunoglobulin A, G, and M levels were normal.

**Table 1:** Serum and CSF markers before and after treatment.

**Test/(range)**	**Pre-treatment**	**Post-treatment**
**Serum**		
IgM RF (<15 IU/mL)	56	18
Anti-CCP (<25 U/mL)	>1,600	706
ILR-2 (158–623 kU/L)	1,065	N/A
**CSF**		
Leukocytes (<5 E6/L)	170	<5
Protein (0.40–0.70 g/L)	1.16	0.28
IgG index (<0,60)	1.45	0.45
Oligoclonal bands	Present	Absent
B lymfocytes (%)	7.80	–
Plasma cells (%)	1.80	–
RF IgM^*^ (<15 IU/mL)	92.7	Negative
Anti-CCP^*^ (<25 IU/mL)	19,600	64
CXCL-13 (<10 ng/L)	>500	<10

* *Range in serum; -, not performed*.

Cerebrospinal Fluid (CSF) analysis revealed a mononuclear pleocytosis (170 E6/L) and elevated protein level (1.16 g/L). Due to the pleocytosis, intravenous ceftriaxone, and aciclovir were administered, to cover for bacterial meningitis and Herpes Simplex Virus (HSV) encephalitis. Subsequent CSF cultures revealed no growth of bacteria, no Borrelia antibodies, and viral/bacterial PCR *(E. coli, hemophilus influenzae, Listeria monocytogenes, Neisseria meningitidis, hemolytic streptococcus, streptococcus pneumoniae, cytomegalovirus, enterovirus, herpes simplex virus, varicella zoster, Cryptococcus, and micromiome 16S/18S*), and flowcytometry, and cytological analysis for malignancy were negative. Therefore, antiviral- and antibiotic- treatment was terminated.

The following days the patient displayed sporadic confusion, delusions, and fever (38.5°C). Subsequent tests, including HIV, syphilis, and tuberculosis were negative. Re-examination of CSF showed continuous mononuclear pleocytosis (130 E6/L), high IgG index (1.45) and presence of oligoclonal bands, suggestive of inflammation. Repeated cultures for bacteria were negative and cytological analysis showed an inflammatory pattern with an elevated number of B-lymphocytes (7.8 %) and plasma cells (1.8%, Table 1).

To investigate possible systemic inflammation or malignancy whole-body FDG-PET CT was performed. This showed hypermetabolism of the cerebral cortex, adjacent to the meningeal enhancement found on MRI, and a right medial lobe infiltrate of the lung. CT of thorax and abdomen confirmed an infiltrate, slight pleural effusion, and pleural thickening. Endobronchial ultrasound with biopsy was performed revealing no malignancy or infection.

On suspicion of RM, we performed analysis on undiluted CSF showing moderately positive IgM RF (92.7 IU/mL) and strongly positive anti-CCP (19,600 IU/mL) and CXCL-13 (>500 ng/L, Table 1).

Subsequent, biopsy of meninges (Figure 1C) confirmed chronic inflammation dominated by CD138 positive plasma cells and a limited number of CD3 positive T-lymphocytes with limited infiltration into the underlying gray matter (Figures 1E,F). Additionally, granulomatous inflammation with dense infiltration of CD68+ histiocytes and the presence of rheumatoid nodules were found (Figures 1D,G). Microbial stains, PCR, and cultures of biopsy tissue for fungi, parasites, acid-fast bacilli, HSV 1, HSV 2, CMV, SV40, M. tuberculosis, and toxoplasmosis were negative.

Based on the (i) MRI findings with patchy meningeal enhancement, (ii) high titer of IgM-RF and anti-CCP in CSF and (iii) histopathological chronic inflammation of meninges with plasma cells and rheumatic nodules, the diagnosis RM was established. Concurrently, the patient displayed extra articular manifestations of RA in her lungs.

Intravenous high dose methylprednisolone (750 + 1,000 + 1,000 mg on three consecutive days) followed by oral tapering was administered in addition to current treatment with methotrexate. Within days symptoms improved, but did not completely resolve. The following weeks, the patient received Rituximab (1,000 mg intravenous, repeated after 14 days). CSF levels of IgM RF, anti-CCP, and CXCL-13 decreased accordingly to the patient reporting significant treatment response (Table 1). A 6 month follow-up MRI showed regression of meningeal enhancement (Figure 1B) and follow-up FDG-PET CT showed almost complete regression of pulmonary findings. Neurological examination at 6 month follow up confirmed resolution of clinical symptoms.

## Discussion

Meningitis in RA is a rare serious extra-articular complication (1, 2, 7, 16). Clinical neurological manifestations are often non-specific and duration and manifestations of RA is unreliable, as less than half of patients display active synovitis (2, 17). Sometimes CNS involvement even precedes the onset of arthritis (17–20). In cases published since 2010, 34% (13 of 38) had no history of RA before the diagnosis of RM (Table A1). CSF findings are variable but most often include a mild pleocytosis with elevated protein concentration and normal glucose (Table A1). Gadolinium enhanced MRI is often useful, showing asymmetrical pachy- or leptomeningeal enhancement (11, 18). Recently, a review of 29 cases of RM showed definite asymmetric meningeal involvement in 62% of patients, and most common neurological features were hemiparesis or hemisensory symptoms mimicking stroke or epilepsy related to localization of meningeal involvement (11). In comparison to this, we find that 70% (33 of 47) had transient or permanent weakness, sensory deficits, or speech disorders, whereas 36% (17 of 47) had seizures (Table A1) It is not uncommon that patients display other extra-articular manifestations of RA such as subcutaneous nodules or pulmonary manifestations, as seen in our case (2, 5, 21, 22).

Patients with RA are often treated with various immunosuppressants which increase the risk of aseptic meningitis or opportunistic infections. Therefore, it is important to rule out iatrogenic aseptic, septic, and fungal diseases before diagnosis of RM. Concurrently, autoimmune diseases, malignancies, other granulomatous diseases or IgG4-related disease can display a similar pattern of dural thickening, making them possible considerations in the differential diagnosis of RM (10, 11, 16).

Until now, there are no known RM biomarkers in CSF and meningeal biopsy is required for definite diagnosis. Biopsy shows thickening of meninges (Figure 1C) and histopathological features include pachy- and leptomeningeal inflammation with plasma cells and the presence of rheumatoid noduli, and in some cases vasculitis (1, 2, 17). Patients diagnosed at autopsy almost all display meningeal rheumatoid noduli, while patients diagnosed with meningeal biopsy most often show non-specific inflammation (2, 7). In some previous cases correlation between strongly elevated CSF RF and Il-6 and RM has been proposed (12, 13, 23, 24), however this still needs validation as a diagnostic tool.

No clear guideline for treatment of RM exists and cyclophosphamide, methotrexate, and azathioprine in combination with corticosteroids have all been described with improvement of symptoms (7, 17, 18, 25). In some cases, improvement on corticosteroid treatment alone has been described (5, 11, 12, 14, 20, 24, 26–31). In our case review 41% (18 of 44) were treated with corticosteroids alone, 2% (1 of 44) received no treatment, whereas the remaining received corticosteroids in combination with another therapy (Table A1). Seven patients (16 %) received rituximab. On these regimens only 1 case worsened (32), 8 (18%) had an incomplete improvement, whereas 80% improved (Table A1).

To our knowledge, anti-CCP in CSF has only been examined in four cases of RM and found to be elevated in three of these (12–15). Serum anti-CCP antibodies help distinguish RA from other types of arthritis, can help to identify patients with a higher risk of severe disease and are rarely found in other autoimmune conditions (33). They are often used in combination with IgM RF in the diagnosis of RA. In this case, anti-CCP level in CSF was strongly positive and a crucial element in both diagnosing RM and monitoring treatment response. With this case, we show a novel clear response of anti-CCP to the treatment of RM. Moreover, in addition to CSF anti-CCP and IgM RF, we also find the B cell chemoattractant CXCL-13 levels associated with treatment response, which to our knowledge has not previously been investigated.

We propose using anti-CCP, IgM RF, and CXCL-13 in CSF as potential biomarkers not only for diagnosis of RM, but also in evaluation of treatment response. Further studies are needed to clarify their potential use.

## Data Availability

All datasets generated for this study are included in the manuscript and/or the Supplementary Files.

## Ethics Statement

Clinical data in this case report was collected with the consent of the patient. A written informed consent was obtained from the patient for the publication of this case report.

## Author Contributions

MN and AN: design and draft of the manuscript and interpretation of data. JF and JM: draft of manuscript. MW and CB: acquisition of data and draft of manuscript. K-EB and TE: revised manuscript for intellectual content. MB: draft of manuscript, acquisition of data, and revised manuscript for intellectual content.

### Conflict of Interest Statement

The authors declare that the research was conducted in the absence of any commercial or financial relationships that could be construed as a potential conflict of interest.

## Appendix

**Table A1:** Summary of RM cases from 2010 to present.

**References**	**Patient (Age, sex)**	**Years of RA**	**Treatment of RA**	**Symptoms of RM**	**Serum**			**CSF**						**MRI compatible with RM**	**Biopsi compatible with RM**	**Treatment**	**Outcome**
					**IgM RF (IU/mL)**	**Anti-CCP (IU/mL)**	**Other**	**Cells/μl**	**Protein (mg/L)**	**Glucose (mg/dl)**	**IgM RF (U/mL)**	**Anti-CCP(IU/mL)**	**Other**				
Cianfoni et al. (32)	74, F	5	CS, MTX	Progressive left-side weakness and hypoesthesia	506	–	–	65	0.43	Normal	–	–	–	Yes	Yes	CS, IT MTX	Worsening
Matsushima et al. (24)	80, F	20	CS, sulfasalazine, bucillamine, etanercept	Transient weakness and numbness right-side	Normal	–	–	18	0.55	–	–	–	IL-6 = 4.6 pg/ml	Yes	Yes	CS	Improvement
Inan et al. (34)	70, F	0	None	Headache, nausea, vomiting, and confusion	108	–	ESR = 124 mm/h	140	1.13	34	98 (after treatment <20)	–	–	Normal	Not performed	CS, AZA	Improvement
Aguilar-Amat et al. (35)	71, F	15	NR	Seizures and PSP-like phenotype	27.9	–	–	Normal	Normal	Normal	–	–	–	Yes	Yes	CS, MTX	Improvement
Kim et al. (26)	66, M	0	None	Seizures (SE) and left-sided weakness	High levels	1,448	ANA high	11	Normal	Normal	–	–	–	Yes	Yes	CS	Improvement
Servioli et al. (36)	80, F	NR	CS, HCQ	Unsteady gait with falls. Progression to left-sided weakness	<20	–	ESR = 35 mm/h	2–7	0.75–0.77	60	–	–	–	Yes	Yes	Not reported	Not reported
Hasiloglu et al. (37)	62, F	4	CS, MTX	Headache, paresis, and paresthesia right UE	351	120	–	40	0.40	–	–	–	–	Yes	Not performed	CS, MTX	Improvement
Huys et al. (38)	58, F	9 month	MTX, Adalimumab	Headache and psychomotor retardation, seizures	–	–	–	30	0.55	–	–	–	–	Yes	Yes	CS, RTX, Leflunomide, MTX d/c, Adalimumab d/c	Improvement
Duray et al. (22)	73, M	1	CS, MTX	Disorientation, apathy, and astenia, walking difficulty	2,720	>340	–	83–91	1.3–2.22	42–58	–	–	–	First MRI normal, yes	Yes	CS, CYC	Improvement
Krysl et al. (5)	62, M	10	HCQ	Epilepsia partialis continua right side	1:320	760	–	0–32	0.245–0.345	–	–	–	OCBs in one CSF sample	Yes	Yes (2 year after initial symtoms)	CS	Improvement
Roques et al. (39)	60, M	NR	MTX	Transient right-sided paresis and hypoesthesia	–	–	–	Increased	Mild elevation	Normal	–	–	–	Yes	Yes	Not reported	Not reported
Hayashi et al. (4)	60, M	10	CS	Parkinsonism not responsive to levo-dopa	–	–	–	13	0.75	Normal	–	–	–	Yes	Yes	CS	Incomplete improvement
Bourgeois et al. (40)	70, M	NR	NR	Transient right hemiparesis, headache	Positive	–	–	68	0.47	2,9 mmol /L	–	–	–	Yes	Yes	CS, HCQ, sulfasalazine	Improvement
Rijkers et al. (6)	57, F	NR	NR	Tonic-clonic seizures	–	–	–	–	–	–	–	–	–	Yes	Yes	CS	Not reported
Yeaney et al. (9)	63, M	9	NR	Headache and paresis	–	–	–	–	–	–	–	–	–	Yes	Yes	Not reported	Not reported
Padjen et al. (20)	77, F	0	None	Seizures and right hemiparesis	171.7	405.3	–	Normal	Normal	Normal	–	–	–	Yes	Yes	CS	Improvement
Lu et al. (27)	60, F	23	CS, Auranofin	Headache, photophobia, insomnia, panic attacks, hallucinations	>1:160	Strongly positive	–	2	0.26	58	–	–	–	Yes	Yes	CS	Improvement
Roy et al. (3)	Late 50s, F	NR	MTX, sulfasalazine	Transient aphasia, confusion, headache right leg weakness, right facial drop	–	High	–	12	0.55	58	–	–	–	Yes	Yes	MTX, MMF, MTX d/c	Improvement
Magaki et al. (28)	37, M	0	None	Headache, facial weakness, speech disorder, right hand dysfunction	83	>250	–	10–16	0.35–0.50	50–89	–	–	–	Yes	Yes	CS	Improvement
Magaki et al. (28)	62, F	0	None	Confusion and transient loss of consiousness, seizures, and lower limb weakness	Negative	–	–	–	–	–	–	–	–	Not reported	Yes	CS	Incomplete improvement
Nihat et al. (41)	71, F	6	Adalimumab, MTX	Dysarthria, paresthesia left face and arm, difficulty walking, tremor, and headache	7,900 U/L	226	ESR = 76 mm/h; ANA 1:80	50–80	0.46–0.67	2.4 mmol/L	–	–	–	Yes	Yes	CS, CYC, MTX	Improvement
Saego et al. (29)	66, F	12	Infliximab	LE numbness, aphasia developing into headache, LE paralysis	–	–	–	213–216	4.4–8.59	41–44	RF elevated	–	–	Yes	Yes	CS	Improvement
Shibahara et al. (12)	63, M	0		Headache, vertigo, confusion	140	472	ESR = 18 mm/h	37	0.92	Normal	–	4.4–26.2	IL-6 = 482 pg/ml	Yes	Not performed	CS	Improvement
Matsuda et al. (21)	66, M	19	CS, MTX, iguratimod	Falls	160	310	ESR = 38 mm/h; ANA 1:5120; SSA and SSB positive	71	1.14	27		–	–	Yes	Not performed	CS, MTX d/c	improvement
Moeyersoons et al. (42)	49, F	N/A	N/A	N/A	N/A	N/A	N/A	N/A	N/A	N/A	N/A	N/A	N/A	N/A	Not performed	CS, RTX. adalimumab d/c, leflunomide d/c	Improvement
Tsuzaki et al. (43)	65, M	7 month	CS, MTX, Entanercept	Transient loss of consciousness, seizures, transient dysrthria, left leg weakness	12	275	sIL2R = 555 U/mL; ANA = 80; SSA 297 U/mL; SSB 18.6 U/mL	12	0.32	55		–	–	First normal, yes	Yes	CS, tocilizumab, etanercpt d/c	Improvement
Choi et al. (11)	65, F	3	CS, MTX, leflunomide	Headache, confusion, and recurrent left hemiparesis	69.3	48.8	–	20	1.134	43	RF 17.6	–	–	Yes	Yes	CS	Improvement
Degboé et al. (44)	59, M	6	MTX	Transient right-sided hypoesthesia and hemiparesis	–	–	–	30	0.75	3.2 mmol/L	–	–	–	Yes	Yes	CS, MTX, RTX	Improvement
Jessee and Keenan(45)	68, F	0	None	Confusion, right-sided weakness, and seizures	208	95.8	ANA 1:640	8	0.65	56	–	–		Not done (pacemaker)	Yes	CS, MTX	Incomplete improvement
Alexander et al. (46)	73, M	NR	Leflunomide	Transient speech disorder, behavoiral change and seizure	45	>340	–	18–100	0.69–1.03	2.5–3.1 mmol/L	–	–		Yes	Yes	CS, RTX	Incomplete improvement
Finkelshtein et al. (19)	66, F	0	None	Headache, transient paresthesia left leg	23–25	266	–	–	–	–	–	–	–	Yes	Yes	None	Improvement
Parsons et al. (47)	76, M	30	MTX	Transient left UE paresis, new onset seizures	Elevated	Elevated	ANA elevated	239	0.39	51	RF negative	–	–	Yes	Yes	CS, MTX	Improvement
Oono et al. (23)	36, F	13	CS, MTX	Headache and transient sensory disturbance right face and UE	–	–	ESR = 56 mm/h; anti-RNP = 15 U/mL	19	0.57	51		–	IL−6 = 843 pg/ml, OCBs	Yes	Not performed	CS, MTX d/c	Improvement
Akamatsu et al. (13)	55, F	6 month	MTX	Speech difficulty, left-sided hemiparesis, and spatial neglect	85 U/L	223.7		68	0.40	52		3.7	IL-6 = 271 pg/mL	Yes	Not performed	CS	Incomplete improvement
Gherghel et al. (10)	77, F	>9 year	Ethanercept, leflunomide	Recurrent speech disorder and left-sided paresthesia and hemparesis	86	119	ANA 1:160	5	0.49	–	–	–	–	Yes	Yes	CS, etanercept d/c, leflunomide d/c	Incomplete improvement
Schuster et al. (14)	48, M	0	None	Headache, recurrent left-sided weakness	298	>340	–	300	1.37	–	–	>340	–	Yes	Not performed	CS	Improvement
Schuster et al. (14)	62, F	Not stated	NR	Recurrent tingling and weakness	146	265	–	Normal		–	–	–	–	Yes	Not performed	CS, MTX	Improvement
Schuster et al. (14)	72, M	0	None	Recurrent sensory motor deficit left-side	133	154	–	51	Normal	–	–	–	–	Yes	Yes	CS	Improvement
Schuster et al. (14)	62, M	11	NR	Alexia, agraphia, acalculia, headache, seizures	22.3	329	–	Normal	Normal	–	–	–	–	Yes	Not performed	CS, tocilizumab	Improvement
Schuster et al. (14)	65, F	11	NR	Recurrent sensory motor deficit left-side, speech disorder	313	26	–	8	0.653	–	–	–	–	Yes	Yes	CS, tocilizumab, leflunomide d/c; MTX d/c	Improvement
Schuster et al. (14)	45, M	30	NR	Recurrent left-side hypoesthesia, headache, ataxia	113	7	–	37	4.6	–	–	–	–	Yes	Yes	CS, CYC, MTX, leflunomide d/c; HCQ d/c	Improvement
Ching et al. (31)	72, F	0	None	Left-sided weakness, psychiatric symptoms, seizures	Negative	197.5	ESR = 39 mm/h	12	0.25	58	–	–	–	Yes	Yes	CS	Improvement
Harrison et al. (48)	53, M	NR	CS, leflunomide, tofacitinib citrate	Headache, seizures, right LE paresis	293	250	ESR 46 mm/h	7	0.64	48	–	–	–	Yes	Yes	CS, RTX	Improvement
McKenna et al. (30)	59, M	0	None	Headache and left-sided weakness, focal onset seizures	88.2	>340	ACE = 70 U/L	Pleocytosis	0.672	3.4 mmol/L	–	–	–	Yes	Yes	CS	Improvement
Pellerin et al. (1)	74, M	3–4	CS, HCQ, MTX	Expressive aphasia, imbalance, potural tremor, parkinsonism, seizures	High	High	ACE 66 U/L, beta 2 mikroglobulin 4,6 mg/L	6	0.86	Normal	–	–	–	Yes	Yes	CS, CYC, MTX d/c	Incomplete improvement
Grose et al. (49)	87, F	NR	None	UE weakness, confusion, hallucinations	143	>200	ANA 1:640	104	1.55	Normal	–	–	–	Yes	Not performed	CS	Incomplete improvement
Scheitel et al. (50)	75, F	9	CS, leflunomide	UE paresthesia, weakness, headache, facial jerks, Rytmic jerks	High	High	ESR = 92 mm/h	14	0.69	–	–	–	–	Yes	Not performed	CS, RTX	Improvement
Lubomski et al. (15)	49, M	0	None	Headache, deterioration in mental state, delusions	8	>600	–	1	0.39	3.4 mmol/l		Strongly positive		Yes	Yes	CS, RTX	Improvement

*ACE, angiotensin converting enzyme; ANA, antinuclear antibodies; AZA, azathioprin; CS, corticosteroids; CYC, cyclophosphamide; d/c, discontinued; ESR, erythrocyte sedimentation rate; F, female; HCQ, Hydroxychloroquine; IL-6, interleukin-6; IT, intrathecal; LE, lower extremity; M, male; MTX, methotrexate; MMF, Mycophenolate mofetil; N/A, not avaliable; NR, not reported; RA, rheumatoid arthritis; RF, rheumatic factor; RM, rheumatoid meningitis; RNP, ribonucleoprotein; RTX, Rituximab; sIL2R, soluble interleukin-2 receptor; SSA, Anti-Sjögren's-syndrome-related antigen A; SSB, Sjögren's-syndrome-related antigen B; UE, upper extremity*.
